# Identifying dynamic regulation with machine learning using adversarial surrogates

**DOI:** 10.1371/journal.pone.0325443

**Published:** 2025-06-05

**Authors:** Ron Teichner, Naama Brenner, Ron Meir

**Affiliations:** 1 Viterbi Department of Electrical and Computer Engineering, Technion - Israel Institute of Technology, Haifa, Israel; 2 Network Biology Research Laboratory, Technion - Israel Institute of Technology, Haifa, Israel; 3 Wolfson Department of Chemical Engineering, Technion - Israel Institute of Technology, Haifa, Israel; Universitat Pompeu Fabra, SPAIN

## Abstract

Biological systems maintain stability of their function in spite of external and internal perturbations. An important challenge in studying biological regulation is to identify the control objectives based on empirical data. Very often these objectives are time-varying, and require the regulation system to follow a dynamic set-point. For example, the sleep-wake cycle varies according to the 24 hours solar day, inducing oscillatory dynamics on the regulation set-point; nutrient availability fluctuates in the organism, inducing time-varying set-points for metabolism. In this work, we introduce a novel data-driven algorithm capable of identifying internal regulation objectives that are maintained with respect to a dynamic reference value. This builds on a previous algorithm that identified variables regulated with respect to fixed set-point values. The new algorithm requires adding a prediction component that not only identifies the internally regulated variables, but also predicts the dynamic set-point as part of the process. To the best of our knowledge, this is the first algorithm that is able to achieve this. We test the algorithm on simulation data from realistic biological models, demonstrating excellent empirical results.

## Introduction

Living systems maintain stability against internal and external perturbations at multiple levels of organization. This phenomenon, known as homeostasis [1–3], is essential for their functioning, and its failure is associated with disease. Typical examples include regulating the temperature and blood sugar level to within certain pre-set limits. Despite their importance, identifying the objectives of homeostatic regulation in complex systems in a *data-driven* way, based on abundant data without a known model, remains a computational and algorithmic challenge.

Control theory, which provides sophisticated tools to model and control dynamical systems [4], is often invoked to describe homeostasis. Yet, it does not directly enable understanding the exact objectives of homeostatic regulation in biological systems. This is due to several reasons. First, control theory is based on a clear separation between the controlled system (or plant) and the controller. In biological systems, the “plant” and “controller” have developed together through evolution, generating a complex network of both positive and negative feedback interactions and cannot be separated [5]. Second, control design is based on a mathematical model for the plant, and deriving such a model for biological systems is not always possible. This highlights the importance of empirical methods to identify biological regulation in a data-driven way.

A related challenge in Machine Learning (ML) is the data-driven identification of conserved quantities in dynamical systems. ML algorithms that address this problem are applicable in different fields of science. A conserved quantity is found in physics, in the context of Hamiltonian dynamics [6]. Epidemiology models (e.g., the Kermack-McKendrick model), as well as population dynamics models (e.g., the Lotka-Volterra model, or the Monod chemostat model), have constants of motion with significant biological implications (see, e.g. [7, 8]). Dedicated ML algorithms have been developed to detect such conserved quantities [9–12], but their applicability to biological systems is limited. For example Inverse Optimal Control and Inverse Reinforcement Learning, that are used in a related context, assume a clear separation between a plant and a controller (see [13, 14] for detailed surveys of both fields). Most algorithmic approaches directed at physical systems search for conserved quantities under the assumption of a given dynamical system with fixed parameters, whereas in biology this is not necessarily the case. Therefore, dedicated algorithmic tools are required to identify the regulated quantities in a data-driven manner in biological systems.

Identifying Regulation with Adversarial Surrogates (IRAS) is a data-driven algorithm for detecting regulated quantities [15]. The basic idea is to find a combination of the measured variables that remains stable as a function of time, and for which shuffling the components of the combination maximally harms this stability. Such a combination is presumably made up of co-varying quantities that compensate for one another. The algorithm iteratively solves a min-max optimization problem by dynamically generating adversarial surrogates. IRAS was verified on systems with known ground-truth, and demonstrated impressive empirical results [15]. It discovered the regulated quantity in examples from different fields including protein interactions, ecological systems, a psychophysical experiment in which a stimulus signal was regulated and physical Hamiltonian systems. Recently, several sufficient conditions guaranteeing local convergence of IRAS were analytically obtained [16].

Algorithms for identifying conserved quantities assume that there exists a function of the observed variables that is constant in time (but may vary between different trajectories). Given a set of measurements over time, $z(t)\in {\mathbb{R}}^{n}$, they attempt to find a real-valued function of the observed variables that is maintained around a fixed set-point:

$$g(z(t))\approx c_{set}\text{ for all }t.$$ (1)

Note that if the function *g* exists, it is not unique. For example, affine and other transformations on *g* are also fixed. Therefore we can assume without loss of generality that $c_{set}=0$. Note also that direct optimization can detect trivial combinations that are not useful. For example, optimizing for conservation alone can (and does) lead to quantities such as a constant function g(z)≡c, independent of *z*. The min-max optimization formulated for IRAS [15] guarantees that the algorithm avoids convergence to such trivial results.

The word homeostasis combines the Greek words *homoios* (“similar”) and *stasis* (“standing still”) yielding the idea of “staying the same”. However, in many biological systems the regulated variables may follow a time-varying function. This is the case for example in systems that are *entrained* to various rhythms like the 24 hours solar day [17–20]. A limitation of many ML algorithms, including IRAS, is that they only identify variables that are regulated around a constant, time-independent, value. Thus, they are incompatible with time-dependent regulation.

Allowing a dynamically changing reference value, contrary to (1), is motivated by biological phenomena such as circadian rhythms in body temperature, sleep-wake cycle regulation, and seasonal changes in fur thickness in animals; well-documented examples include the Baroreflex control of the cardiovascular system, where slow blood-pressure and heart-rate oscillations are observed [21], neuronal oscillations in brain activity, which are crucial for cognition and motor control [22], and menstrual cycles of hormones like estrogen, which regulate ovulation and menstruation [23].

In this work, we consider systems that regulate an unknown function of the observables around an unknown dynamically changing reference value,

g(z(t))≈c(t). (2)

We do not assume any specific form for either the regulated function *g*() or the setpoint process *c*().

The IRAS algorithm searches for a function that is regulated about a fixed set-point (1). Inferring meaningful *dynamic* regulatory processes, (2), renders the task impossible for IRAS to solve. Here we generalize IRAS to allow inferring meaningful dynamic regulatory processes, as is required in many biological settings. We present Identifying Dynamic Regulation with Adversarial Surrogates (IDRAS), a purely data-driven algorithm that simultaneously learns these two separate unknowns, a function *g*() and the dynamic process that it follows, *c*(). This removes a crucial obstacle in the detection of regulation in biological systems, which exhibit sustained oscillations and other modulations of their homeostatic set-points. We expect this algorithm to be widely applicable to biological measured data at multiple levels of organization, and to contribute to revealing their regulatory logic.

The remainder of this paper is organized as follows. Section ‘Algorithm development’ details the new IDRAS algorithm, and Section ‘Examples’ validates its performance on several simulated datasets with a known control objective. The final section concludes and describes possible directions for further research. Throughout the manuscript, we interchangeably refer to the regulated quantity as either a function of the observables or a combination of the observables. When referring to a function that remains constant over time, we also describe it as a conserved quantity.

## Algorithm development

IDRAS is an iterative algorithm consisting of two competing players. The input to the algorithm is an observed sequence of measurements, *z*(*t*), and the goal is to identify both a function of these observables, *g*(*z*(*t*)), and a dynamic reference value *c*(*t*) (2). Since we assume that *g*(*z*(*t*)) follows the reference *c*(*t*), it should be possible, given the observations *z*(*t*) for $t_{0}\leq t\leq t_{1}$, to predict a future value *g*(*z*(*t*_2_)), where $t_{2}>t_{1}$. Intuitively, to detect the coupled pair g(·) and c(·), one could straightforwardly minimize the prediction error, but this may lead to a trivial solution such as g(z(t))=const. To overcome this difficulty, the IDRAS algorithm iteratively optimizes a quantitative measure that characterizes the sensitivity of the prediction error to destroying the temporal order of the observed time-series. It is expected that a regulatory process which involves co-variation among variable would be sensitive to their temporal order, while a trivial constant would not.

**Fig 1:**
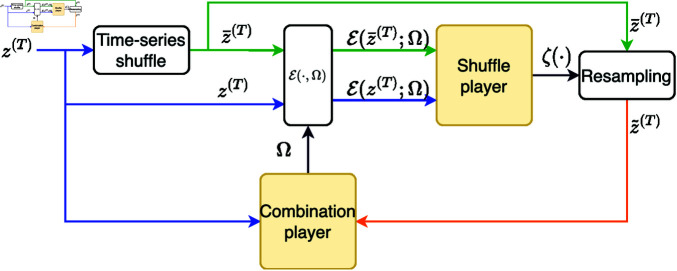
IDRAS algorithm outline. The observation time-series *z* is permuted according to (8) to create the unconstrained series $\bar{z}$. The shuffle player, only exposed to the 1*D* filtering errors, sets the resampling function ζ() used to resample $\tilde{z}$ from $\bar{z}$ such that the distributions of filtering errors are identical, (10). Then the combination player, given *z* and $\tilde{z}$, updates the parameters Ω towards minimizing the filtering error variance ratio, (12). These steps continue to iterate until no further improvement is possible. The block ℰ(·;Ω), based on (5) and whose architecture is detailed in Fig 2, replaces the function *g*() in Fig 3 of [15].

The algorithm iterates between two players as seen in Fig 1. The first, the *Combination player*, iteratively minimizes the error, while the other adversarial *Shuffle player*, successively creates more constrained shuffled ensembles of the data. We call these ensembles "adversarial surrogates", since the second player aims to render the task of the first player more difficult by forcing it to extract information about the temporal structure of the data, which is absent from surrogate data created by random time-shuffling. upon convergence, the algorithm outputs a coupled pair - the function *g*(*z*(*t*)) and the dynamics *c*(*t*) that it follows. This allows to assess the significance of the identified function. We next present a detailed mathematical formulation of the problem and the IDRAS algorithm.

### Problem formulation

We are interested in identifying empirically, from a set of measured observables, a function of these observables which tightly follows a dynamically changing reference value, where both are unknown. The function could represent an internal quantity of high importance to the system, and the reference value could reflect temporal trends in the environment. The input to the algorithm is a sequence ${\left\{z^{k}\right\}}_{k=1}^{N}$ of observations where typically $z^{k}\in {\mathbb{R}}^{n}$ is the noisy output of a continuous-time dynamical system measured at time kΔt. We would like to identify two parametric functions. The first, $g:{\mathbb{R}}^{n}\times {\mathbb{R}}^{b_{\theta }}\to \mathbb{R}$, is such that

$$c^{k}=g(z^{k};\theta ),$$ (3)

is the time-series of the (learned) regulated quantity. We assume that this time-series follows some unknown dynamics and that it is possible to learn a filter, namely a predictor of the value of *c*^*k*^ based on its previous values (formally (4) below is a 1-step predictor, but, following [24], we refer to it as a filter). This leads to our second learned function (predictor), formally given by

$${\hat{c}}^{k}=F(c^{k-T:k-1};\Theta ).$$ (4)

The structure of the learned filter *F*, and the meaning of its parameters, is detailed at the end of the present Section, and $c^{k-T:k-1}=[c^{k-T},c^{k-T+1},\ldots ,c^{k-1}]$ with *T*>0 a hyper-parameter. The filtering error is,

$$e^{k}:=c^{k}-{\hat{c}}^{k}\overset{\Delta }{=}ℰ(z^{k-T:k};\Omega ),$$ (5)

and is a function of the parameters

Ω=[θ,Θ] (6)

where $ℰ:{\mathbb{R}}^{n(T+1)}\times {\mathbb{R}}^{b_{\Omega }}\to \mathbb{R}$ such that


$$ℰ(z^{k-T:k};\Omega )=g(z^{k};\theta )-F(c^{k-T:k-1};\Theta )$$

and $b_{\Omega }=b_{\theta }+b_{\Theta }$. The goal is to converge to a parameter vector $\Omega^{*}$ such that the error is small, namely $e^{k}\approx 0$ for all *k*. We assume that the parametric functions are neural networks and Ω is the vector of parameters in these networks. We note that a straightforward optimization yields the trivial pair, g()≡0, F()≡0, implying the need to formulate a different optimization problem that avoids this trivial solution.

The problem formulated is in fact a generalization of the static problem (1). It is immediate to verify that by setting F(·;Θ)≡0, we obtain $ℰ(z^{k-T:k};\Omega )=g(z^{k};\theta )$, $e^{k}=c^{k}$ and thus we optimize to find a function g(·;θ) such that $c^{k}\approx 0$, namely regulated around a fixed value. This simpler problem was addressed in [15] and in [16] where several sufficient conditions guaranteeing local convergence of IRAS were derived. The next Section introduces the generalization of this algorithm to time-varying regulation set-points.

### Identifying dynamic regulation with adversarial surrogates

IDRAS is based on the probabilistic assumption that the noisy sample series {*z*^*k*^} is the output of a stationary process and thus admits a probability density function (PDF)


$$f_{z}:{\mathbb{R}}^{n}\to {\mathbb{R}}_{+}$$

implying that the sequence *z*^*k*−*T*:*k*^ admits a PDF


$$f_{z^{\left(T\right)}}:{\mathbb{R}}^{n(T+1)}\to {\mathbb{R}}_{+}$$

for all *T*>0. We denote the random variable of a length-*T* + 1 sequence by


$$z^{\left(T\right)}\sim f_{z^{\left(T\right)}}$$

where $z^{\left(T\right)}\in {\mathbb{R}}^{n(T+1)}$. Then the filtering error is a scalar projecting of *z*^(*T*)^ by ℰ(·,Ω), whose (scalar) PDF $f_{z^{\left(T\right)}}^{\;\Omega }:\mathbb{R}\to {\mathbb{R}}_{+}$ is

$$f_{z^{\left(T\right)}}^{\;\Omega }(e):=\int_{\left\{q|ℰ(q;\Omega )=e\right\}}f_{z^{\left(T\right)}}(q)\;dq,$$ (7)

where e∈ℝ and $P(e_{1}\leq ℰ(q;\Omega )\leq e_{2})=\int_{e_{1}}^{e_{2}}f_{z^{\left(T\right)}}^{\;\Omega }(e)\;de$. To denote the variance of the filtering error we will use the notation $var(f_{z^{\left(T\right)}}^{\;\Omega })$ and, in general, for any PDF $f:{\mathbb{R}}^{n(T+1)}\to {\mathbb{R}}_{+}$ we denote the PDF [variance] of its projection by ℰ(·;Ω) with $f^{\;\Omega }:\mathbb{R}\to {\mathbb{R}}_{+}$ [$var(f^{\;\Omega })$].

Straightforward minimization of $var(f_{z^{\left(T\right)}}^{\;\Omega })$ will usually lead to a trivial solution $\Omega^{*}$ (F=g≡0) as mentioned earlier. Therefore, to find a non-trivial $\Omega^{*}$, IDRAS also uses a surrogate PDF


$$f_{{\bar{z}}^{\left(T\right)}}:{\mathbb{R}}^{n(T+1)}\to {\mathbb{R}}_{+},$$

that corresponds to a random variable


$${\bar{z}}^{\left(T\right)}=[{\bar{z}}^{\left(T-1\right)},\bar{z}]$$

and is defined

$$f_{{\bar{z}}^{\left(T\right)}}({\bar{z}}^{\left(T\right)})=f_{z^{\left(T-1\right)}}({\bar{z}}^{\left(T-1\right)})f_{z}(\bar{z}).$$ (8)

In the right hand side we see that $f_{{\bar{z}}^{\left(T\right)}}$ is the PDF for the case where a sequence of observations ${\bar{z}}^{\left(T-1\right)}$ of length *T* is followed by a random observation $\bar{z}$ drawn from the marginal distribution. Clearly this ‘detached’ observation $\bar{z}$, or any non-trivial function of it $g(\bar{z};\theta )$, cannot be predicted given ${\bar{z}}^{\left(T-1\right)}$ with a mean-square-error lower than ${var}_{z\sim f_{z}}(g(z;\theta ))$. Therefore, given the structure of $f_{{\bar{z}}^{\left(T\right)}}$, to learn a meaningful (g,F) pair it seems a natural idea to find a vector Ω that minimizes the ratio of filtering error variances,

$$\frac{var(f_{z^{\left(T\right)}}^{\;\Omega })}{var(f_{{\bar{z}}^{\left(T\right)}}^{\;\Omega })}.$$ (9)

While (9) eliminates the trivial solution g()≡0 which will give a ratio of 0/0, it fails to eliminate all trivial solutions. Consider the case of analyzing a time-series of vital physiological signals where $z^{k}=[{BP}^{k},{HR}^{k}]$ are the values of blood-pressure and heart-rate sampled every second. Let *T* = 1. Clearly, all the samples $z^{\left(1\right)}\sim f_{z^{\left(1\right)}}$, where $z^{\left(1\right)}=[z^{0},z^{1}]$ is the concatenation of two sequential samples, are plausible sequential cardiovascular measurements. These samples admit, among other constraints, that $|{HR}^{1}$ − ${HR}^{0}|<M_{HR}$, namely, the rate of change of heart-rate cannot exceed a maximal value $M_{HR}$ [${\text{sec}}^{-1}$]. A sample ${\bar{z}}^{\left(1\right)}\sim f_{{\bar{z}}^{\left(1\right)}}$ drawn from the surrogate PDF, where the samples are not sequential, may violate this physiological constraint. Such an artefact, which is an implausible physiological observation, can superficially increase the denominator in (9), in turn leading to an erroneous solution. We refer to such a solution as trivial since it highlights a feature in the data that is a biological constraint rather than the result of a regulatory process (see Discussion for further details on this). To address this difficulty, IDRAS iteratively eliminates non-plausible observations in the surrogate PDF. In what follows we mathematically formalize the case of a trivial, artefact solution.

**Remark 1.** *Assume that there exists a set $D\subseteq {\mathbb{R}}^{n(T+1)}$ such that*


$$\int_{D}f_{{\bar{z}}^{\left(T\right)}}(q)\;dq>0,\text{ and }\int_{D}f_{z^{\left(T\right)}}(q)\;dq=0$$

*then any vector Ω for which $ℰ(z^{\left(T\right)};\Omega )=0$ for all $z^{\left(T\right)}\notin D$ and $ℰ(z^{\left(T\right)};\Omega )\neq 0$ for $z^{\left(T\right)}\in D$, minimizes the ratio in (9) - achieving a value zero. This is usually a "trivial", or artifact, solution that reflect constraints in the data; however a straightforward optimization algorithm minimizing ratio (9), may converge on such a solution.*

IDRAS is an iterative algorithm consisting of two competing players, a generalization of the IRAS algorithm. The players solve a min-max style optimization problem with respect to the ratio of filtering error variances.

**Shuffle player:** Given the current solution $\Omega^{i-1}$, update the surrogate PDF $f_{{\bar{z}}^{\left(T\right)}}$ to a surrogate PDF $f_{{\tilde{z}}^{\left(T\right)}}:{\mathbb{R}}^{n(T+1)}\times {\mathbb{R}}^{b_{\Omega }}\to {\mathbb{R}}_{+}$ by

$$f_{{\tilde{z}}^{\left(T\right)}}({\tilde{z}}^{\left(T\right)};\Omega^{i-1}):=f_{{\bar{z}}^{\left(T\right)}}({\tilde{z}}^{\left(T\right)})\zeta \left(ℰ({\tilde{z}}^{\left(T\right)};\Omega^{i-1});\Omega^{i-1}\right),$$ (10)

where $\zeta :\mathbb{R}\times {\mathbb{R}}^{b_{\Omega }}\to {\mathbb{R}}_{+}$ is a resampling function that is chosen such that the modified surrogate PDF satisfies

$$f_{{\tilde{z}}^{\left(T\right)}}^{\;\Omega^{i-1}}(p;\Omega^{i-1})=f_{z^{\left(T\right)}}^{\;\Omega^{i-1}}(p)\text{ for almost all }p\in \mathbb{R}.$$ (11)

The goal of the shuffle player is thus to transform $f_{{\bar{z}}^{\left(T\right)}}$ to a PDF $f_{{\tilde{z}}^{\left(T\right)}}$ such that the statistical properties of the filtering errors of the random variables *z*^(*T*)^ and ${\tilde{z}}^{\left(T\right)}$ are identical. This overcomes the difficulty described in Remark 1. A closed-form expression for ζ is given in Lemma 1 below.

**Combination player:** Set the parameters vector to minimize the ratio of filtering error variances,

$$\Omega^{i}:=\underset{\Omega }{\text{argmin}}\frac{var(f_{z^{\left(T\right)}}^{\;\Omega })}{\text{var}(f_{{\tilde{z}}^{\left(T\right)}}^{\;\Omega }(\Omega^{i-1}))}.$$ (12)

The shuffle player is “adversarial” w.r.t the combination player because (11) implies that the ratio


$$\frac{var(f_{z^{\left(T\right)}}^{\;\Omega })}{\text{var}(f_{{\tilde{z}}^{\left(T\right)}}^{\;\Omega }(\Omega^{i-1}))}{|}_{\Omega =\Omega^{i-1}}=1$$

in contrast to its minimization in (12). The two players mutually inform each other of their current step results, and the process continues iteratively until the combination player can no longer decrease the ratio in (12). We refer to this algorithm as IDRAS and depict its outline in Fig 1.

**Lemma 1.** *The function ζ guaranteeing that (11) holds is given by*

$$\zeta (x;\Omega )=\left\{ \begin{array}{cc} f_{z^{\left(T\right)}}^{\;\Omega }(x)/f_{{\bar{z}}^{\left(T\right)}}^{\;\Omega }(x), & \text{ if }f_{{\bar{z}}^{\left(T\right)}}^{\;\Omega }(x)\text{⧸}=0,\\ 0, & otherwise. \end{array}\right.$$ (13)

*Furthermore, this ζ also guarantees that $f_{{\tilde{z}}^{\left(T\right)}}({\tilde{z}}^{\left(T\right)};\Omega^{i-1})$ in (10) is indeed a PDF.*

For the proof of Lemma 1 we refer the reader to the proof of Lemma 1 in [16] with the following modifications: θ←Ω, $z\leftarrow z^{\left(T\right)}$ and g←ℰ. For example $f_{\tilde{z}}^{\theta }$ is replaced by $f_{{\tilde{z}}^{\left(T\right)}}^{\;\Omega }$.

#### Filter.

The architecture of the filter within the block ℰ(·;Ω) in Fig 1 has many degrees-of-freedom and can be chosen by the user according to prior knowledge regarding the nature of the dynamic reference. To impose a minimal number of constraints on the filter F(·) it can be implemented by a fully connected deep neural-network.

**Fig 2:**
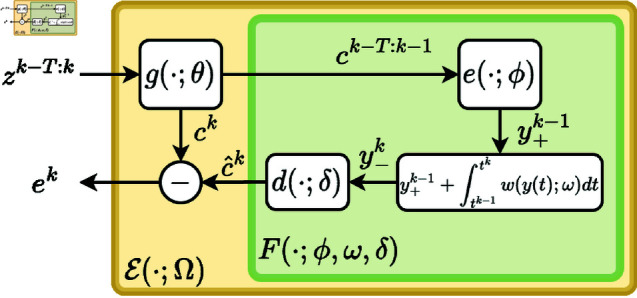
Architecture of the ℰ(·;Ω) block (5) from Fig 1. The filtering error *e*^*k*^ is the difference between the learned reference value *c*^*k*^ and its prediction ${\hat{c}}^{k}$. The filter F(·;Θ), (14) with Θ=[ϕ,ω,δ], infers a latent-state $y_{+}^{k-1}$ and time-advances it to the state $y_{-}^{k}$ from which the estimation ${\hat{c}}^{k}$ is decoded.

Dealing with biological systems, we assume that the dynamics of the reference can be modeled by a continuous-time, time-invariant latent model (see [25, 26] for details on integrating differential equations using neural-networks). Our filter-block contains three parts: *(i)* An encoder $e:{\mathbb{R}}^{T}\;\times \;{\mathbb{R}}^{b_{\phi }}\to {\mathbb{R}}^{b_{y}}$, that given *T* consecutive values of the reference, *c*^*k*−*T*:*k*−1^, infers a latent state $y_{+}^{k-1}$ (*b*_*y*_ a user-defined hyper-parameter), *(ii)* A drift function $w:{\mathbb{R}}^{b_{y}}\;\times \;{\mathbb{R}}^{b_{\omega }}\to {\mathbb{R}}^{b_{y}}$ describing the deterministic term in the dynamics of the latent state that serves to time-advance the latent state $y_{+}^{k-1}$ to $y_{-}^{k}$, *(iii)* An emission function $d:{\mathbb{R}}^{b_{y}}\;\times \;{\mathbb{R}}^{b_{\delta }}\to \mathbb{R}$ that decodes the 1-step predicted value ${\hat{c}}^{k}$ from the latent state $y_{-}^{k}$. The following set of equations describe the filter (4),

$$y_{+}^{k-1}=e(c^{k-T:k-1};\phi ),\;y_{-}^{k}=y_{+}^{k-1}+\int_{t^{k-1}}^{t^{k}}w(y(t);\omega )dt,\;{\hat{c}}^{k}=d(y_{-}^{k};\delta ),$$ (14)

depicted, as part of the ℰ(·;Ω) block in Fig 2 and where Θ=[ϕ,ω,δ]. Concluding the presentation of IDRAS, we note that by construction (see Eq (8)) IDRAS is deliberately incapable of detecting stationary control objectives and therefore it is not a substitute for IRAS. The two algorithms query for fundamentally different control objectives and should be evaluated independently, as we demonstrate in the following examples. See the S1 Appendix for a detailed mathematical explanation.

#### Multiple time-series.

Often, multiple time-series are observed from a system or from similar systems. The algorithm can be applied to multiple time-series without any modifications. In the description above, we simply replace *z*^*k*^ by *z*^*m*,*k*^ which represents the ${\text{k}}^{\text{th}}$ sample of the ${\text{m}}^{\text{th}}$ time-series, and is distributed according to *f*_*z*_. Likewise, $c^{m,k}=g(z^{m,k};\theta )$, ${\hat{c}}^{m,k}=F(c^{m,k-T:k-1};\Theta )$ and $e^{m,k}=c^{m,k}\;-\;{\hat{c}}^{m,k}=ℰ(z^{m,k-T:k};\Omega )$. We note that the algorithm processes multiple time-series concurrently in a single execution that results in a single solution $\Omega^{*}$.

## Examples

After presenting the construction of IDRAS, we seek to validate it on datasets with a known control objective, so that the quality of the results can be assessed. To assess the performance, we calculate the Pearson correlation $\rho (c^{*},c)$ between the known control objective ${c^{*}}^{k}$ and the output of IDRAS *c*^*k*^. In addition, we calculate $err(c,\hat{c})$, the normalized-mean-square-error of the learned filter given by


$$err(c,\hat{c})\overset{\Delta }{=}\frac{var(c-\hat{c})}{var(c)}.$$

Below we present two validation examples using models of biological dynamic processes: kinetics of protein interactions, and bacterial life cycle. In each model there is a known control objective, detected by IDRAS. In S1 Appendix we present validation of a synthetic example containing two independent (and therefore uncorrelated) control objectives; running IDRAS several times shows that in each evaluation it successfully converges to one of the control objectives and not to some combination of the two, which is an undesired result. The architecture of all parameterized functions and the values of all the example model parameters are listed in S1 Appendix. Also please find in S1 Appendix the terms for ρ() and err() for multiple time-series processing. Code reproducing the results is available at https://github.com/RonTeichner/IRAS.

### A kinetic model of regulated gene expression

Circuits and networks of interacting proteins and other cellular components are thought to take part in control mechanisms inside the cell [27]. Specifically, the regulation of gene expression can be modeled at a coarse-grained level by kinetic equations, where continuous variables represent concentrations of participating proteins, mRNA or other molecules, and their interactions are formulated in mass-action approximation [28].

**Fig 3:**
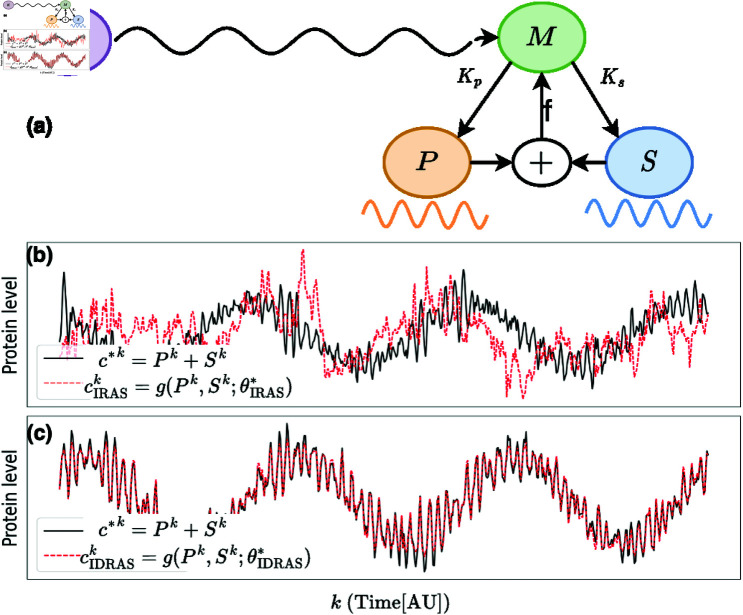
Validation of IDRAS on an oscillating setpoint in a kinetic model. (a) Illustration of the regulated gene expression feedback-loop model. mRNA *M*, produced at a rate *K*(*t*), induces the production of proteins *S* and *P* and receives a negative feedback of their sum. Since biochemical interactions are faster than mRNA production, this circuit regulates the sum *S* + *P* to follow its time course of production *K*(*t*) (see Eq 17). (b,c) Comparison of algorithm outputs (dashed red) to the known regulated combination *P*  +  *S* (black). (b) The IRAS algorithm does not converge, since it searches for a constant regulation set-point that does not exist in this model (the depicted $c_{\text{I}RAS}^{k}$ is not stable and continues to change with further iterations of IRAS). (c) The IDRAS algorithm captures with high precision the control objective and its oscillating trend.

Our first example focuses on a model that describes the production of two proteins whose sum is maintained near a setpoint by a feedback loop (inspired by [29]). An mRNA molecule *M* is transcribed at a rate *K* and degraded at a rate $\gamma_{M}$. This molecule in turn determines the production rate of two proteins, *P* and *S*. The total amount of two proteins *P* and *S*, namely *P* + *S*, feeds back to affect the level of *M*. Fig 3a illustrates the kinetic interactions model. The mRNA *M*, and the two proteins *P* and *S*, are linked in a feedback loop; the strength of this negative feedback given by the rate constant *f*. In the limit of strong feedback, the combination *P* + *S* is maintained around a set-point proportional to *K*.

This model, with a fixed *K* resulting in a fixed set-point, was used as a validation for the IRAS algorithm in previous work [15]. Here, we incorporate a time-dependent setpoint: we assume that the external environment modulates transcription rate, resulting in an oscillatory setpoint *K*(*t*). The model is described by the stochastic differential equations

$$\;dM=(K(t)-f(P+S)-\gamma_{M}M)\;dt,\;\;dP=(k_{P}M-\gamma_{P}P)\;dt+\eta_{P}\;dW_{P}(t),\;\;dS=(k_{S}M-\gamma_{S}S)\;dt+\eta_{S}\;dW_{S}(t),$$ (15)

where *W*_*P*_(*t*) and *W*_*S*_(*t*) are Wiener processes that were added to the kinetic scheme to represent various sources of noise in the biological system. The rate is given by

$$K(t)=K_{0}(1+0.5\cos(2\pi t/\tau_{K}+\phi_{K})).$$ (16)

We further assume that the typical timescale of environmental change is slower than that of the biochemical kinetic interactions. For example circadian cycles occur over 24 hours whereas protein production and degradation - over minutes. Under these conditions of timescale separation, the feedback loop operates faster than the modulations in transcription rate. Small changes in *S* or *P*, modeled by increments of the Wiener processes *W*_*P*_ and *W*_*S*_, induce swift and sharp changes in the transcription of *M* and maintain P+S around a reference level

$$c^{*}(t)\overset{\Delta }{=}(P+S)(t)=\frac{1}{f+\frac{\gamma_{M}\gamma_{P}\gamma_{S}}{k_{P}\gamma_{S}+k_{S}\gamma_{P}}}K(t),$$ (17)

extending the result for a fixed setpoint *K* by following its slow variations *K*(*t*). The indirect interactions between the two proteins is reflected in a negative covariation of *S* and *P*, here relative to the slowly-modulated setpoint. Our observables contain the levels of the two proteins *P* and *S*,


$$z^{k}=[P^{k},S^{k}]=[P(t=kt_{s}),S(t=kt_{s})],$$

where 1ts is the sampling rate.

To demonstrate the performance of IDRAS and compare it to IRAS, we simulated multiple time-series (for ease of notation we drop the time-series index *m* throughout the example) of (15) and ran both IRAS and IDRAS algorithms in search of the control objective, obtaining the solutions $\theta_{\text{I}RAS}^{*}$ and $\Omega_{\text{I}DRAS}^{*}$ (containing $\theta_{\text{I}DRAS}^{*}$, see (6)) respectively. Fig 3b depicts the output (dashed red) $c_{\text{I}RAS}^{k}=g(z^{k};\theta_{\text{I}RAS}^{*})$ trained by the IRAS algorithm. Due to the lack of a regulated constant combination, IRAS did not converge and the time-series $c^{*}$ and *c* have a Pearson correlation value of *ρ*_I*RAS*_(c*,c)=0.21. Fig 3c similarly depicts the output of IDRAS, $c_{\text{I}DRAS}^{k}=g(z^{k};\theta_{\text{I}DRAS}^{*})$, showing that the combination P+S was precisely found, despite its oscillating nature with a Pearson correlation value of *ρ*_I*DRAS*_(c*,c)=0.99.

When running IDRAS, the normalized-mean-square-error can also be applied to the quality of prediction. A score of ${\text{err}}_{\text{I}DRAS}(c,\hat{c})=0.14$ was obtained, indicating that not only was the correct combination found, but also that its temporal modulation is well predicted.

### Bacterial life cycle

The next example we consider is a realistic biological model of bacterial growth homeostasis, where growth and division proceed for many generations with significant variability and statistical stability. This is a problem with a long history but is still at the focus of much current research. We apply our algorithm to simulation data, which well mimic experimental measurements but where the regulation is known.

Most bacteria grow smoothly with their size accumulating exponentially, and divide abruptly [30–32]. This behavior is consistent with a threshold crossing by some division indicator; specifically three distinct indicators corresponding to different regulation modes have been studied: cell size (“sizer” control mechanism), added size (“adder” mechanism) and elapsed time (“timer”) [33, 34]. More generally, phenomenological models can interpolate continuously between these three types [32, 35, 36]. It is common practice to identify the regulation mode by empirically observing the correlation of a quantity at the end of the cell cycle - size, added size or time - with its initial value. The intuition behind this heuristic is that if some quantity triggers division when crossing a threshold, it should appear uncorrelated with the initial value.

As in most threshold processes in biology, regardless of what is the indicator - the threshold for division is not expected to be strictly fixed, but to fluctuate over time. In a recent paper, Luo *et al*. [37] demonstrate that the heuristic approach based on correlation plots can only uncover the correct mode of regulation under the restricted condition of a fixed threshold. However, an alternative method to identify the division indicator, which takes into account threshold dynamics, was not offered. Below we simulate the dynamics of bacterial growth and division with a realistic "sizer" mechanism - namely, division occurs when cell size reaches a fluctuating threshold. We show that the IRAS algorithm, designed to detect fixed set-points, performs poorly in the presence of threshold dynamics. In contrast, IDRAS accurately identifies both the cell-division mechanism and the threshold dynamics, resulting in an excellent prediction of the time series.

The threshold *u*(*t*) is modeled by a stochastic Ornstein-Uhlenbeck process with a characteristic timescale $\tau_{u}$,

$$\;du=\frac{\mu_{u}\;-\;u}{\tau_{u}}\;dt+\sqrt{2\frac{\sigma_{u}^{2}}{\tau_{u}}}\;\;dW(t),\;t>0$$ (18)

with initial condition $u(0)\sim 𝒩(\mu_{u},\sigma_{u}^{2})$ and where *dW*(*t*) is an increment of a Wiener process. We assume that within the ${\text{k}}^{\text{th}}$ cycle the cell size *x*(*t*) grows exponentially at a rate $\alpha_{k}$, until it reaches the threshold size and divides at time $t_{d}^{k}$ by a factor $\eta_{k}$. The equations describing these growth and division processes are:

$$x(t)=x_{b}^{k}e^{\alpha^{k}(t-t_{b}^{k})},\;t_{b}^{k}\leq t\leq t_{d}^{k}\;x_{b}^{k}=x(t_{d}^{k-1})\eta^{k},\;t_{b}^{k}=t_{d}^{k-1}\;t_{d}^{k}={argmin}_{t}\{t\geq t_{b}^{k}|x(t)=u(t)\},$$ (19)

where $x_{b}^{k}\overset{\Delta }{=}x(t_{b}^{k})$ is the birth size, $x_{d}^{k}\overset{\Delta }{=}x(t_{d}^{k})$ is the size at division. The exponential growth rate $\alpha^{k}$ is found in experiment to be randomly distributed across cycles [31, 38]; therefore we assume in our model it is a random variable drawn independently each cycle from a Gamma distribution: *α*^k^~Γ(*γ*_shape_,*γ*_scale_). Symmetric division with added Gaussian noise implies that $\eta^{k}\sim 𝒩(0.5,\sigma_{\eta }^{2})$ is the division fraction. The initial condition for the simulation is taken as $x_{d}^{0}\sim 𝒩(\mu_{u},\sigma_{u}^{2}),t_{d}^{0}=0$.

**Fig 4:**
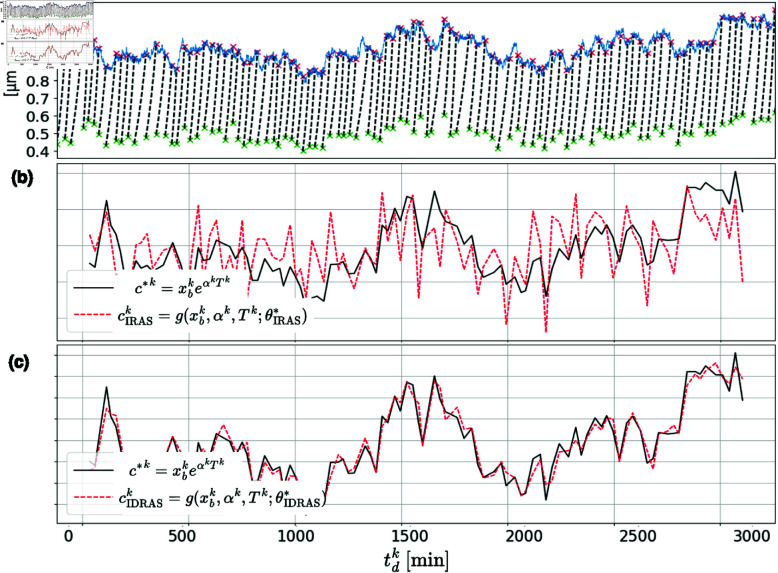
Validation of IDRAS on a model of bacterial growth and division. (a) Bacteria life-cycles. Cell size increases continuously during each cell cycle (black curve) and divides approximately in half at discrete division events. See (19). Division occurs when cell size crosses the stochastic threshold process *u*(*t*), (18) (blue curve). Birth size and division size are indicated by green and red marks respectively. (b) IRAS does not decouple the cell-size mechanism (black-curve) from the threshold trend *c*(*t*) and yields a function (dashed-red) that represents their mixture. (c) IDRAS captures the division-mechanism.


We simulated these dynamics of cell size over multiple cycles of growth and division and over multiple lineages (for ease of notation, we drop the lineage index *m* throughout the example). Here division regulation is known and follows the *sizer* mechanism - the cell divides when its size crosses the dynamic threshold *u*(*t*). Fig 4a depicts one such simulated lineage over time - the cell-size, *x*(*t*) (dashed-black), and the stochastic threshold *u*(*t*) (blue). Our observables, derived from *x*(*t*), contain the initial size, growth rate and the cycle duration, namely

$$z^{k}=[x_{b}^{k},\alpha^{k},T^{k}=t_{d}^{k}-t_{b}^{k}]$$

where $\alpha^{k}=(1/T^{k})\log(x_{d}^{k}/x_{b}^{k})$. We note that our choice of the observables *z*^*k*^ renders the identification task harder, as now to correctly detect the sizer mechanism the network has to learn the function $g(z)=x_{b}e^{\alpha T}$ and not just *g*(*z*) = *x*_*d*_, in the case *x*_*d*_ was an entry of *z*_*k*_.

We ran both IRAS and IDRAS algorithms in search of the division control mode. We chose a realistic parameter set for which heuristic identification methods based on data correlations fail [37]. Fig 4b depicts the output (dashed red) $c_{\text{I}RAS}^{k}=g(x_{b}^{k},\alpha^{k},T^{k};\theta_{\text{I}RAS}^{*})$ trained by the IRAS algorithm as a function of time over many cycles, together with the ground-truth output, the size threshold $x_{b}e^{\alpha T}$ (black line). Here IRAS, aiming for a combination regulated around a fixed setpoint (see (1)), outputs a function that results from fusing together the division indicator and the dynamic threshold *u*(*t*), yielding a relatively low correlation between its output and the ground truth combination: *ρ*_I*RAS*_(c*,*c*)=0.614.

In contrast to other algorithms that try to fit a conserved quantity to a combination of known functions, IRAS is data-driven and model independent. Therefore, to understand the meaning of the resulting output of the neural network some intuition and knowledge about the problem needs to be employed. To interpret the result of IRAS, we note that our model simulations are performed in the regime where threshold fluctuations are significantly slower than the single-cycle time scale, namely $\tau_{u}\gg T$. In this regime the threshold is approximately constant along a single-cell cycle, $u(t)\approx u(t_{b}^{k})$ for $t_{b}^{k}\leq t\leq t_{d}^{k}$. The birth size, about half the size at division of the previous cell is thus $x_{b}^{k}\approx 0.5u(t_{b}^{k})$, and the size at division is $x_{d}^{k}\approx u(t_{b}^{k})$. These approximations allow us to write the regulated quantity as the difference between final size and threshold, and express it with size variables:

$$x_{b}^{k}e^{\alpha^{k}T^{k}}-u(t_{b}^{k})\approx x_{b}^{k}e^{\alpha^{k}T^{k}}-2x_{b}^{k}\approx 0,$$ (20)

a mixture of two terms. The first, $x_{b}^{k}e^{\alpha^{k}T^{k}}$, represents the size threshold while the second, $-2x_{b}^{k}$, is an influence of the dynamics of the threshold process.

IDRAS, in contrast, is designed to decouple the two quantities - division indicator and dynamic threshold - by separately learning the threshold dynamics and a function which is regulated w.r.t. this dynamic reference value (see (2)). Fig 4c depicts $c_{\text{I}DRAS}^{k}=g(x_{b}^{k},\alpha^{k},T^{k};\theta_{\text{I}DRAS}^{*})$, demonstrating the precise identification of the division control mode with a Pearson correlation value of *ρ*_I*DRAS*_(c*,*c*)=0.97, where ${c^{*}}^{k}=x_{b}^{k}e^{\alpha^{k}T^{k}}$ (in comparison, the correlations of the adder mechanism for which ${c^{*}}^{k}=x_{b}^{k}(e^{\alpha^{k}T^{k}}-1)$ and timer mechanism, ${c^{*}}^{k}=T^{k}$, are 0.74 and 0.1 respectively). The normalized-mean-square-error is ${\text{err}}_{\text{I}DRAS}(c,\hat{c})=0.23$, very close to the expected value of 0.25 derived from (18) and (19). To establish the latter value, note that the average cell has a growth rate of $\bar{\alpha }:=\gamma_{\text{s}hape}\gamma_{\text{s}cale}$ (the mean value of a Gamma distribution) and, from simple stability constraints one can conclude that it grows by a factor of 2. Therefore, its mean cell-cycle is approximately


$$\bar{T}:={\bar{\alpha }}^{-1}\log2.$$

Consider a cell-cycle that begins at time *t* and has an average duration of $\bar{T}$. The variance of $u(t\;+\;\bar{T})$ given *u*(*t*) is known to be $\sigma_{u}^{2}(1\;-\;e^{-2\frac{\bar{T}}{\tau_{u}}})$ (see Chapter 4.4.4 in [39]) and since the variance of *u*(*t*) is $\sigma_{u}^{2}$ we conclude that


$${\text{err}}_{\text{I}DRAS}(c,\hat{c})=1-e^{-2\frac{\bar{T}}{\tau_{u}}},$$

if indeed IDRAS has converged to the solution


$$c^{k}=g(z^{k};\theta_{\text{I}DRAS}^{*})=x_{d}^{k}=u(t_{d}^{k}).$$

Substituting the model parameters yields $1-e^{-2\frac{\bar{T}}{\tau_{u}}}=0.25$ up to 2 digit precision.

We conclude that in this example, judging by either measures of performance, IDRAS successfully detected the division indicator in the presence of a dynamic threshold. This task is already known to be unsolvable by heuristic methods, and an alternative identification method was not previously suggested.

Finally, we comment on relations to real experimental data. Modern experiments that integrate microfluidics techniques and advanced imaging analysis enable direct measurements of growth and division cycles within a lineage over extended periods, as simulated in this study. Observations and data collection are conducted across various laboratories [31, 32, 38, 40–46]. These datasets provide a valuable test-bed for evaluating different hypotheses regarding regulatory mechanisms. In practical applications of the algorithm to real-world data, predefined hypotheses can be compared to the output of g(·) as demonstrated here. Alternatively, a comprehensive analysis of the input-output relationship of the trained network g(·) should be conducted as a complementary task following an IDRAS run. This will be further elaborated in the Discussion Section.

## Discussion

Detecting regulated or conserved quantities in dynamic data is a technically challenging problem with many potential applications. Recently, the IRAS algorithm was introduced [15, 16]. The algorithm receives as input raw dynamic measurements and provides functions of the observables that are maximally conserved across time. Building on this advancement, we here presented IDRAS, an algorithm capable of identifying control objectives that are regulated with respect to a *dynamic* reference value. This removes a crucial obstacle in applying such identification algorithms to detect regulation in biological systems, which exhibit sustained oscillations and other modulations of their homeostatic set-points.

Using models with known ground truth control, we presented empirical results demonstrating that IDRAS can simultaneously identify the control objective - a combination of the observed variables that is regulated - and predict its dynamics. Being a purely data-driven method, our approach explains the system’s behavior in the ‘language’ of the observables, without prior assumptions. On the other hand, this approach is obviously constrained by the measurements; the underlying assumption is that with a large number of measurements, control objectives can be well approximated by their combination. Upon completion of an IDRAS run, the algorithm provides the normalized-mean-square-error, a performance metric indicating how closely the system adheres to the identified dynamical set point.

The control objective is represented by the function implemented by the trained network, g(·). In contrast to other approaches, the network does not confine the result to a pre-defined class of functions but rather captures a huge range of possibilities. The drawback is that the network provides the output function without a closed-form equation or interpretation of the result. Therefore, analyzing the function of g(·) is required following an IDRAS run in order to provide such an interpretation. This analysis can be performed manually by fixing certain inputs and examining the effects of others on the output, or automatically using Symbolic Regression tools, which aim to derive an analytical expression of the network’s functionality [47] (see example B Eq 14 in [15]).

The data-driven approach incorporated in IRAS and IDRAS is not limited to a small number of variables, and this is a strength relative to other methods where combinatorics explode and make identification implausible. However, for very large system dimensionality, interpretation of the resulting combination can be difficult. Moreover, It can be argued that tracking of a setpoint in a high-dimensional system does not necessarily imply the existence of a control mechanism within the system - A set-point can result from the mutual interactions within the biological environment. Such a phenomenon aligns with the concept of distributed control, where autonomous controllers are dispersed throughout the system [48, 49], and a global control objective emerges. Another possibility is that, in a system with multiple feedback loops, a potentially meaningful combination will be detected which is not a control objective *per se;* it can nevertheless represent a meaningful compound quantity that may help shed light on the system’s functionality.

What can we learn about biological control in cases where dimensionality is too high, or direct interpretation is difficult for other reasons? one can simply plot the output of the learned function g(·) across time, and observe its characteristic timescale. This observation can potentially provide important information on the system’s behavior, under the assumption that it encodes a quantity of importance. An additional characteristic of the system that can be determined directly from the IDRAS output, is the effective memory time, derived from the hyper-parameter *T*. As time progresses, the impact of previous states on the current state of the system diminishes. Consequently, increasing *T* beyond the effective memory time does not improve the detection and inference results. Researchers are advised to conduct multiple iterations of IDRAS, incrementing *T* until no further improvement in the normalized-mean-square-error is observed. The final value of *T* thus represents the system’s effective memory time.

While our algorithm offers to be useful for analyzing a broad range of empirical data, we emphasize that such an analysis is not expected to be completely automatic and should always be accompanied by some understanding of the biological system. For example, the result can be compared to a-priori hypotheses generated by other more heuristic methods; competing hypotheses can be compared to one another to find the one most consistent with the data. In addition, caution should be taken as spurious correlations between measurements may result in artefactual regulated combinations.

Topics for further research include a rigorous analysis of the algorithm to prove convergence, equilibrium points and stability. We expect this algorithm to be widely applicable to experimental biological measurement at multiple levels of organization, and to contribute to revealing their regulatory logic.

## Supporting information

S1 Appendix Please find the Appendix in the IDRAS_Appendix.pdf file
(PDF)
