# Supplementary material for: Identifying dynamic regulation with machine learning using adversarial surrogates
Source: PLoS One. 2025 Jun 5;20(6):e0325443. doi: 10.1371/journal.pone.0325443 (PMC12140394; doi:10.1371/journal.pone.0325443)
Supplement: S1 Appendix — Please find the Appendix in the IDRAS_Appendix.pdf file (PDF) [file pone.0325443.s001.pdf]

Identifying Dynamic Regulation with Machine Learning using Adversarial Surrogates - Supporting Information

S1 Appendix.

Architecture and performance metrics (Section Examples)

All the parameterized functions are feed-forward artificial neural networks [1] with an input layer of 32 neurons, a hidden layer of 16 neurons and an output layer of appropriate dimension. The activation function of all neurons is Leaky-ReLU except for the output neuron whose activation function is the Sigmoid function. The hyper-parameters are $T = 10$ and $b_y = 2$ and we train over 200 epochs using the Adam optimizer with a learning rate of 0.001. IRAS runs are performed with the parameters detailed in [2].

The Pearson correlation coefficient between two timeseries $x^{m,k}$ and $y^{m,k}$, averaged over M pairs of time-series when processing multiple time-series, is defined as:

$$\rho(x,y) = \frac{1}{M}\sum_{m=1}^{M}\frac{\sum_{k=1}^{n}(x^{m,k}-\bar{x}^{m})(y^{m,k}-\bar{y}^{m})}{\sqrt{\sum_{k=1}^{n}(x^{m,k}-\bar{x}^{m})^2\sum_{k=1}^{n}(y^{m,k}-\bar{y}^{m})^2}},\ \bar{\alpha}^{m} := \frac{1}{n}\sum_{k=1}^{n}\alpha^{m,k}.$$

The normalized-mean-square-error for the case of multiple (M) time-series is defined,

$$\text{err}(c,\hat{c}) = \frac{1}{M}\sum_{m=1}^{M}\frac{\text{var}(c^m-\hat{c}^m)}{\text{var}(c^m)},$$

where c^m and $\hat{c}^m$ are the random variables obtained for time-series m.

A kinetic model of interactions (Section Kinetic model of regulated gene expression)

The timescale of the feedback loop $\tau_{(P+S)} = {}^1\!/_f = 0.0005 (F = 2000)$ is much shorter than the timescale of environmental influenced oscillations in K, $\tau_K = 0.2$. We simulated a dataset of 100 observed systems sampled at rate ${}^1\!/_{t_s} = 1$ with $K_s = K_p = 150$, $\gamma_S = \gamma_P = 70$, $\gamma_M = 80$, $f = 2000$, $K_0 = 300$ and $dW_P, dW_S \sim \mathcal{N}(0,(0.5dt)^2)$. For each system $\phi_K \sim \mathcal{U}[0,\pi]$ and $M(0), P(0), S(0) \sim \mathcal{U}[0.02, 0.1]$. For this setting of the parameters we obtained $\rho_{\text{IDRAS}}(c^*, c) = 0.99$. Challenge the algorithm by increasing the frequency of the dynamical set point by 150% we obtain $\rho_{\text{IDRAS}}(c^*, c) = 0.897$. We note that the algorithm runs a single time over the multiple observed time-series as described in the end of the Section describing the algorithm in the main text.

Bacterial life cycle (Section Bacterial life cycle)

The simulated dataset contains 100 lineages each consisting of 100 generations. In (18) $\mu_u = 1\,[\mu\text{m}]$, $\sigma_u = 0.1\,[\mu\text{m}]$ and $\tau_u = 200\,[\text{min}]$; Initial conditions are $u(0) \sim N(\mu_u, \sigma_u^2)$ for each lineage. In (19) the growth rate is sampled from a Gamma-distribution, $\alpha^k \sim \Gamma(25, 9.4\cdot 10^{-4})$ and the division fraction is distributed $\eta^k \sim \mathcal{N}(0.5, 0.05^2)$. The initial conditions for the first cell in each lineage are $x_b^0 \sim \mathcal{N}(0.5, 0.05^2)$, $t_b^0 = 0$. We note that the algorithm runs a single time over the multiple observed lineages as described in the end of the Section describing the algorithm in the main text.

The constraints on IDRAS vs IRAS by the surrogate data

While potentially, as explained below, IDRAS is capable of predicting stationary control objectives similarly to IRAS, these are distinct tasks. Consequently, IDRAS has been purposely constructed to detect only dynamic setpoints. Therefore, unlike IRAS, IDRAS does not identify stationary setpoints.

At its core, IDRAS searches for differences between (the distribution of the projection by Ω, see Eq. (7)) a length-T sequence of the observed data,

$$z^{(T)} \sim f_{z^{(T)}},$$

and a sequence of the surrogate data,

$$f_{\bar{z}^{(T)}}(\bar{z}^{(T)}) = f_{z^{(T-1)}}(\bar{z}^{(T-1)}) f_z(\bar{z}^T),$$

see Eq. (8), where $\bar{z}^{(T)} = [\bar{z}^{(T-1)}, \bar{z}^T]$. For the sake of argument, assume that $f_{z^{(T)}}(z^{(T)}) = \prod_{k=0}^{T} f_z(z^k)$, such that only a stationary relation exists (if at all). Under this assumption, using Eq. (8) we obtain the surrogate data

$$\begin{aligned} f_{\bar{z}^{(T)}}(\bar{z}^{(T)}) &= f_{z^{(T-1)}}(\bar{z}^{(T-1)}) f_z(\bar{z}^T) \\ &= \prod_{k=0}^{T-1} f_z(\bar{z}^k) f_z(\bar{z}^T) \\ &= \prod_{k=0}^{T} f_z(\bar{z}^k), \end{aligned}$$

which equals the distribution of the observed data, $f_{\bar{z}^{(T)}}(\bar{z}^{(T)}) = f_{z^{(T)}}(\bar{z}^{(T)})$. Since the two distributions are identical, IDRAS will not find any control objective in the case where only a stationary one exists. In contrast, the surrogate PDF defined in IRAS is the marginal PDF of the single-variable PDFs product,

$$\bar{z} \sim f_{\bar{\bar{z}}}(\bar{z}) = \prod_i f_{\bar{\bar{z}}_i}(\bar{z}_i),$$

where $f_{\bar{\bar{z}}_i}(\bar{z}_i) = \int_{\{q|q_i=\bar{z}_i\}} f_z(q)\, \mathrm{d}q$ and $\bar{z}_i$ denotes the ith entry of $\bar{z}$. This surrogate PDF differs from the PDF of the observed data when a stationary control objective exists and therefore allows IRAS to detect stationary control objectives. Instead of the definition in Eq. (8), we could have defined the surrogate data of IDRAS as,

$$f'_{\bar{z}^{(T)}}(\bar{z}^{(T)}) = f_{z^{(T-1)}}(\bar{z}^{(T-1)}) f_{\bar{\bar{z}}}(\bar{z}^T),$$

such that the surrogate data now differs from the PDF of the observed data also when a stationary control objective exists. To see this we again assume that $f_{z^{(T)}}(z^{(T)}) = \prod_{k=0}^{T} f_z(z^k)$, but now we obtain the surrogate data

$$\begin{aligned} f'_{\bar{z}^{(T)}}(\bar{z}^{(T)}) &= f_{z^{(T-1)}}(\bar{z}^{(T-1)}) f_{\bar{\bar{z}}}(\bar{z}^T) \\ &= \prod_{k=0}^{T-1} f_z(\bar{z}^k) f_{\bar{\bar{z}}}(\bar{z}^T) \\ &\neq f_{z^{(T)}}(z^{(T)}), \end{aligned}$$

Yet, noting that it renders the task computationally more difficult, since we enlarged the space of possible solutions, with no advantage over separately running the two algorithms, we chose the surrogate data of Eq. (8).

We conclude that by construction IDRAS is incapable of detecting stationary control objectives and therefore it is not a replacement for IRAS. The two algorithms query for fundamentally different control objectives and should be evaluated independently.

Multiple control objectives

To further challenge the IDRAS algorithm, we evaluate it on two examples from the recently published "Feynman Symbolic Regression Database" (FSReD) [3]. This database is composed of a variety of nonlinear equations **inspired** by the physics of electromagnetic fields, that can be used as benchmarks for algorithm performance. Here, we simulate these equations with some combination of parameters following a well-defined time variation, to test the ability of IDRAS to decouple oscillations from the quantities they follow. In addition we concatenate the observables of the two physical systems into a single vector and demonstrate that IDRAS, when fed with the concatenated vector, successfully converges to one of the observed control objectives. This enables the analysis of systems with multiple observed control objectives.

In the first example, we consider a charged particle in an electric field and a time-varying magnetic field: the force F experienced by the particle is

$$F = q \cdot (E_f + Bv\sin(\omega t)),$$

where q is its electric charge, v its velocity, E_f the electric field and B the magnetic field that varies sinusoidally with time with an angular frequency ω. We note that in general, forces, velocities, and fields are vector quantities. Here we consider a case where (in a Cartesian coordinate system (x, y, z)) the magnetic field has a component only in the y-direction and the velocity has a component only in the x-direction. Consequently, the resulting magnetic force is directed solely in the z-direction, leading to the one-dimensional equation presented above.

We simulate 100 time-series of length 100, each rotating at a fixed angular velocity ω. For each time sample z^k, the observables (q, E_f, B, v) are sampled uniformly in $[1, 5]$. Although not physically valid, it is the way the FSReD dataset is synthesized [3]. We expect IDRAS to identify the combination

$$g(F, q, E_f, B, v; \theta^*) = (Bv)^{-1}\left(\frac{F}{q} - E_f\right) = \sin(\omega t) = c_1^*(t).$$

In the second example we consider a nonlinear response,

$$x_{\text{out}}(t) = K(x_{\text{in}}(t) + x_{\text{in}}^2(t)),$$
$$x_{\text{in}}(t) = \cos(\omega' t),$$

where for example $x_{\text{in}}(t)$ might be a current in a system and $x_{\text{out}}(t)$ the voltage. To quantify the performance of IDRAS, we compute $\rho(c^*, c)$ for each of the two chosen examples from the fields of electric-field forces ($I.12.11$) and non-linear responses ($I.50.26$) in Table S1. The example numbers are taken from [3] based on [4]. As shown in Table S1, the agreement is excellent. We are not aware of any other algorithm capable of identifying arbitrary oscillating combinations. In biological systems with a very high number of variables, one may expect a situation where two or more varying control objectives are followed. To examine this scenario, we combine the observations

Table S1. IDRAS captures physical relations

	Equation	$\rho(c^*, c)$
$I.12.11$	$F = q * (E_f + Bv\sin(\omega t))$	0.954
$I.50.26$	$x_{\text{out}} = K(\cos(\omega' t) + \cos^2(\omega' t))$	0.976

from two independent systems into a single vector,

$$z = [F, q, Ef, B, v, \tilde{x}_{\text{out}}, K],$$

and assess IDRAS using these concatenated observations. Here $\tilde{x}_{\text{out}}$ is a noisy version of x_{out} and the introduced noise in $I.50.26$ serves to test whether IDRAS will converge to the noiseless relation, $I.12.11$. The observations now contain two independent oscillatory functions and we test whether IDRAS converges to one of the functions or, to a combination of the two, which is an undesired result. The implication of convergence to one of the functions is highly important as it enables the correct analysis of an observed biological system with multiple controls.

Intuitively, since IDRAS searchers for a combination whose 1-step prediction has a low error, and the prediction error of a combination of multiple independent functions always has a higher error than that of predicting one of them, we expect IDRAS to converge to the identification of a single control objective.

We ran IDRAS 40 times on the data of concatenated observations and analyzed the 10 runs with the lowest normalized-mean-square-error. Let $c_1^*(t)$ and $c_2^*(t) = \frac{\tilde{x}_{\text{out}}}{K} = \cos(\omega' t) + \cos^2(\omega' t)$ be the ground-truth control objectives of equations $I.12.11$ and $I.50.26$ respectively, and $c_r = g(z; \theta_r^*)$ the output of IDRAS at run $r = [1, 2, \ldots, 10]$. For run r, the control objective that best correlates with the output of IDRAS is given by

$$i_r^* := \underset{i \in \{1,2\}}{\operatorname{argmax}} \, \rho(c_i^*, c_r),$$

and its score is $s_r^* := \rho(c_{i_r^*}^*, c_r)$. Likewise the control objective that least correlates with the output of IDRAS is given by $\bar{i}_r^* := \operatorname{argmin}_{i \in \{1,2\}} \rho(c_i^*, c_r)$ and the corresponding score is $\bar{s}_r^* := \rho(c_{\bar{i}_r^*}^*, c_r)$.

The mean values (standard-deviations) of s_r^* and $\bar{s}_r^*$ over the ten runs are $\frac{1}{10}\sum_{r=1}^{10} s_r^* = 0.9318(\pm 0.0686)$ and $\frac{1}{10}\sum_{r=1}^{10} \bar{s}_r^* = 0.2485(\pm 0.2225)$ respectively, testifying that one control objective is well identified while the other is rejected. In addition, in five out of the ten runs we obtain $i_r^* = 1$ (and thus $i_r^* = 2$ in the other five), testifying that IDRAS randomly converges to one of the control objectives contained within the observations.

References

1. Zhang A, Lipton ZC, Li M, Smola AJ. Dive into Deep Learning; 2020.

2. Teichner R, Shomar A, Barak O, Brenner N, Marom S, Meir R, et al. Identifying regulation with adversarial surrogates. Proceedings of the National Academy of Sciences. 2023;120(12):e2216805120.

3. Udrescu SM, Tegmark M. AI Feynman: A physics-inspired method for symbolic regression. Science Advances. 2020;6(16):eaay2631.

4. Feynman RP, Leighton RB, Sands M. The feynman lectures on physics; vol. i. American Journal of Physics. 1965;33(9):750–752.
